# *Polypodium leucotomos* targets multiple aspects of oral carcinogenesis and it is a potential antitumor phytotherapy against tongue cancer growth

**DOI:** 10.3389/fphar.2022.1098374

**Published:** 2023-01-05

**Authors:** Pammela A. Lacerda, Luan C. Oenning, Guilherme Cuoghi Bellato, Lucilene Lopes-Santos, Natalícia de Jesus Antunes, Bruno Augusto Linhares Almeida Mariz, Gabriela Teixeira, Rafael Vasconcelos, Gustavo Ferreira Simões, Ivani Aparecida de Souza, Clóvis Antônio Lopes Pinto, Tuula Salo, Ricardo D. Coletta, Taize M. Augusto, Carine Ervolino de Oliveira, Nilva K. Cervigne

**Affiliations:** ^1^ Laboratory of Molecular Biology and Cell Culture (LBMCC), Faculty of Medicine of Jundiaí (FMJ), Jundiaí, Brazil; ^2^ Laboratory of Pharmacology, University of Campinas, Campinas, Brazil; ^3^ Department of Oral Diagnosis, School of Dentistry, University of Campinas, Piracicaba, Brazil; ^4^ Department of Morphology, Faculty of Medicine of São Paulo Santa Casa, São Paulo, Brazil; ^5^ Department of Physiology, Faculty of Medicine of Jundiaí (FMJ), São Paulo, Brazil; ^6^ Graduate Program in Health Sciences, Faculty of Medicine of Jundiaí (FMJ), São Paulo, Brazil; ^7^ Department of Morphology and Basic Pathology, Faculty of Medicine of Jundiaí (FMJ), São Paulo, Brazil; ^8^ Department of Oral and Maxillofacial Diseases, University of Helsinki, Helsinki, Finland; ^9^ Graduate Program in Oral Biology, School of Dentistry, University of Campinas, Piracicaba, Brazil; ^10^ Department of Internal Medicine, Faculty of Medicine of Jundiaí (FMJ), São Paulo, Brazil; ^11^ Department of Pathology and Parasitology, Universidade Federal de Alfenas (UNIFAL), Alfenas, Brazil; ^12^ Graduate Program in Biological Science, Universidade Federal de Alfenas (UNIFAL), Alfenas, Brazil

**Keywords:** *Polypodium leucotomos*, polyphenolic extract, antitumor effect, immune-inflammation, OSCC progression, EMT-epithelial to mesenchymal transformation

## Abstract

**Introduction:** Oral cancer refers to malignant tumors, of which 90% are squamous cell carcinomas (OSCCs). These malignancies exhibit rapid progression, poor prognosis, and often mutilating therapeutical approaches. The determination of a prophylactic and/or therapeutic antitumor role of the polyphenolic extract *Polypodium leucotomos(PL)* would be relevant in developing new tools for prevention and treatment.

**Methods:** We aimed to determine the antitumor effect of PL by treating OSCC cell lines with PL metabolites and evaluating its action during OSCC progression *in vivo*.

**Results:** PL treatment successfully impaired cell cycling and proliferation, migration, and invasion, enhanced apoptosis, and modulated macrophage polarization associated with the tumoral immune-inflammatory response of tongue cancer cell lines (TSCC). PL treatment significantly decreased the expression of MMP1 (*p* < 0.01) and MMP2 (*p* < 0.001), and increased the expression of TIMP1 (*p* < 0.001) and TIMP2 (*p* < 0.0001) in these cells. The mesenchymal-epithelial transition phenotype was promoted in cells treated with PL, through upregulation of E-CAD (*p* < 0.001) and reduction of N-CAD (*p* < 0.05). PL restrained OSCC progression *in vivo* by inhibiting tumor volume growth and decreasing the number of severe dysplasia lesions and squamous cell carcinomas. Ki-67 was significantly higher expressed in tongue tissues of animals not treated with PL(*p* < 0.05), and a notable reduction in Bcl2 (*p* < 0.05) and Pcna (*p* < 0.05) cell proliferation-associated genes was found in dysplastic lesions and TSCCs of PL-treated mice. Finally, N-cad(Cdh2), Vim, and Twist were significantly reduced in tongue tissues treated with PL.

**Conclusion:** PL significantly decreased OSCC carcinogenic processes *in vitro* and inhibited tumor progression *in vivo*. PL also appears to contribute to the modulation of immune-inflammatory oral tumor-associated responses. Taken together, these results suggest that PL plays an important antitumor role in processes associated with oral carcinogenesis and may be a potential phytotherapeutic target for the prevention and/or adjuvant treatment of TSCCs

## Introduction

Oral squamous cell carcinoma (OSCC) is the ninth leading cause of death in terms of mortality rate of malignant neoplasms, representing an important health problem worldwide(Bray et al., 2018). OSCCs have a highly variable clinical course, and due to their late diagnosis in advanced stages and poor response to therapy, the overall survival rate is less than 50% in 5 years(Koyfman et al., 2019; Zanoni et al., 2019). Thus, new therapeutic options for OSCC are needed to improve patient survival and decrease morbidity(Lippman et al., 2018).

Plants have been proven to be an important natural source for anticancer therapy for several years (Caccialanza et al., 2007; Abdal Dayem et al., 2016; Berman et al., 2016). Recent evidence has shown promising results with natural polyphenols for the prevention and treatment of different types of cancers, including lung, gastric, colorectal, breast, and prostate cancers (Zhou et al., 2016). Polyphenols are part of an extensive category of compounds found in several natural sources, meaning that they should be present in a balanced and healthy diet. Thus, polyphenols have been considered potential candidates for the discovery of anticancer drugs.

*Polypodium leucotomos* (PL) is a species of fern, polyphenol enriched, originating in Central America and also known as *anapsos*. Plant extracts are primarily obtained from the root (rhizome), but secondarily from the leaves of the fern species, with associated nootropic, immunomodulatory, anti-inflammatory, antioxidant and antitumor activities. It can be used as raw material for topical and oral formulations. Together, these compounds seem to favorably attenuate the course of immunological diseases and are a potent antioxidant widely used as an oral photoprotector. The herbal medicine also has anti-inflammatory properties, cytokine suppressor and leukotriene inhibitor. There are studies that also indicate that its use associated with Vitamin E or C could guarantee an even more effective result (Garcia et al., 2006; Antonio De Oliveira Batistuzzo et al., 2021). PL extracts have a known composition of polyphenols, among which are 4-hydroxycinnamic (p-coumaric), 3-methoxy-4-hydroxycinnamic (feluric), 3,4-dihydroxycinnamic (caffeine), 3-methoxy-4-hydroxybenzoic (vanylic) and 3- caffeoyl (chlorogenic) (Caccialanza et al., 2007). It is also known that its antioxidant capacity is dose-dependent, with the most potent among those listed above being ferulic and caffeic acids (Gombau et al., 2006). Notably, when metabolized, polyphenols give rise to phenolic derivatives (Gombau et al., 2006), which are secondary metabolites of many plants, including the one we focused on in this study. Lewandowska et al. (2013) (Lewandowska et al., 2013) demonstrated that despite the rapid absorption of phenolic derivatives, they are metabolized slowly by the body, leading to products with more polar characteristics and active metabolites. Some studies have also provided evidence of its ability to modulate the action of reactive oxygen species and factors related to inflammation and interact with estrogen receptors, directly interfering in reducing the invasiveness of cancer cell lines. (Kim and Park, 2013; Bhatia, 2015; Yoshimaru et al., 2015).

The first evidence of the effect of PL on cancer came from a study by Rodríguez-Yanes et al. (2012) (Rodríguez-Yanes et al., 2012), who demonstrated UV-induced epidermal cell proliferation and enhanced p53 expression and plasma antioxidant capacity in hairless mice. Later, using different approaches Rodríguez-Yanes et al. (2014) (Rodríguez-Yanes et al., 2014) showed that oral administration of PL delayed skin tumor development in UV-irradiated hairless mice. However, little is known about the biological mechanisms underlying the antitumor effects of PL in cancer. Previous investigation of our group showed that PL treatment led to modulation of viability, proliferation and apoptosis, and regulation of migration and invasion, through extracellular matrix (ECM) remodeling and epithelial-mesenchymal transition processes in skin cancer cells *in vitro* (data not published yet). The EMT and ECM stiffness processes have also been highlighted to have a key relevance in oral cancer evolution, so they have gained a great potential not only as potential molecular biomarkers for diagnosis and prognosis, but also to assist further investigations for new therapeutical targeted-drugs. As the involvement of PL as a potential phytotherapeutic tool in oral cancer progression has not yet been investigated, and there is a good well-stablished *in vivo* model to determine its potential antitumor effect, we sought to investigate PL role in oral carcinogenesis.

In this study, we evaluated the modulation of several tumorigenic processes by treatment with PL metabolites in OSCC cell lines with different malignant potential. Regarding the need for information focusing on the chemo-preventive potential of naturally occurring compounds, the current study was also conducted to evaluate the role of PL treatment on 4NQO-induced tongue carcinogenesis. The *in vitro* findings were confirmed using the *in vivo* oral carcinogenesis model. Moreover, to gain insight into the molecular mechanisms by which PL affects the cell cycle, epithelial-mesenchymal transition, and extracellular matrix (ECM) remodeling, OSCC cell lines treated and untreated with PL metabolites were analyzed for major genes associated with migration, invasion, and metastasis. Treatment with PL resulted in a decrease in cell cycling, proliferation, migration, and invasion of OSSC cells and an increase in apoptosis. PL treatment also promoted mesenchymal-epithelial transition phenotypes, such as positive modulation of E-cadherin (E-CAD), negative regulation of vimentin (VIM), and reduced production of matrix metalloproteinases 1 (MMP1) and 2 (MMP-2). These results suggest that PL treatment plays an important role in the modulation of tumorigenic processes associated with OSCCs progression, and may be a potential target for prophylactic or adjuvant oral cancer therapy.

## Material and method

### Cell culture

Human oral cancer cell lines SCC-9, SCC-15, and SCC-25, originally isolated from a 25-, 55-,and 70-year-old male patients with tongue squamous cell carcinoma, respectively, were obtained from American Type Culture Collection (ATCC) and cultured as recommended in a 1:1 mixture of Dulbecco**’**s modified Eagle**’**s medium and Ham**’**s F12 medium (DMEM/F12; Invitrogen, United States) supplemented with 10% fetal bovine serum (FBS), 400 ng/ml hydrocortisone (Sigma-Aldrich, United States), and antibiotics. All three OSCC lines were derived from aggressive tumors, with SCC-15 being slightly less than the others. LN-1 SCC-9-ZsGreen-derived oral cells, isolated from metastatic cervical lymph nodes (Agostini et al., 2014) were cultured in the same medium. The human keratinocyte cell line derived from histologically normal skin (HaCat) and the non-transformed human gingival keratinocyte cell line (HGK), obtained from mucosal keratinocytes and found to be spontaneously immortalized, were used for the cytotoxic assay (control). HaCat was cultured in RPMI1640 medium (Invitrogen, United States) supplemented with 10% FBS and antibiotics, and HGK was cultured in serum-free, low calcium media (Gibco’s Keratinocyte-SFM; Invitrogen, United States) containing specific supplements and antibiotics, as previously described (Mäkelä et al., 1998; Rodrigues et al., 2017). The cells were cultured at 37°C in a humidified atmosphere containing 5% CO_2_.

### *Polypodium leukotomos* metabolites

The standardized dry extract used in our study was acquired already processed of the Modherma Pharmacy (Valinhos, SP, Brazil), from the supplier Pharmanostra. It is an extract obtained from the root (rhizome) of the fern species *Polypodium leucotomos* (Polypodiaceae). Briefly, the PL dry pure extract was initially in a methanol stock solution (1 g/ml), diluted to a S2 solution (40 mg/mL), followed to a pre-metabolized S3 solution (1.6 mg/mL), which was incubated with 1 mL of microsome solution, so that this would guaranteed a standardized final metabolite solution (S4) (0.8 mg/ml). 1.6 mg/ml PL solution was incubated with 2.0 mg/mL of microsomes from Sprague-Dawley rat liver (Animal Facility Center at the State University of Campinas, CEMIB-UNICAMP; CEEA protocol nº 4120-1), 7mM MgCl_2_, 100 mM potassium phosphate buffer (pH 7.4) and 10 µM dapaconazole, in a total volume of 100 µl, for 5 min at 37°C in a water bath with agitation. Then, 100 µl of the NADPH Regeneration System solution (0.5 mM NADP, 10 mM glucose-6-phosphate and 1U glucose-6-phosphate dehydrogenase in 100 mM PBS + 5 mM sodium citrate) was added to start the reactions. The samples were then incubated for a optimized metabolization time of 20 min. The enzymatic reaction was stopped by the addition of 400 µl cold acetonitrile, vortexed for 30 s, and centrifuged at 3000 g at 4°C for 10 min to precipitate the precipitated protein. The supernatant was dried under air flow and frozen at −70°C, until further use. (Pearce et al., 1996; Jia et al., 2010; Marques et al., 2014). Considering that the quantitative composition of a plant extract can vary from one batch to another and in order to minimize differences in the process, a same pre-metabolized S3 solution batch was used for the given optimized incubation time (20 min) to generate the standard metabolite solution, which was then divided into smaller aliquots and freezy-dried to facilitate storage, handling and use during the *in vitro* experiments. Just immediately before use, these aliquots were thawed on ice, brought to room temperature, and diluted in concentrations for application to cell cultures (0.25, 2.5, 25, 250, and 500 ug/ml).

### Treatments with *Polypodium leucotomos* metabolites

The *PL* metabolites, containing the phenolic derivatives of metabolism, were dissolved in a culture medium, aliquoted, and stored at −80°C. To assess the effect of PL, cells were cultured in growth media containing 0.25, 2.5, 25, 250 or 500 ug/ml for 24 and 48 h.

### Apoptosis analysis

The apoptosis index was determined using annexin V-FITC and 7-AAD labeling (BD Biosciences, United States) as previously described(Rodrigues et al., 2022). A minimum of 10,000 events were analyzed for each sample using a FACSCalibur flow cytometer equipped with an argon laser (BD Biosciences).

### MTT assay

Cell viability was evaluated using MTT (tetrazolium blue thiazol-3-[4,5-dimethyl-thiazol-2-yl]-2,5-diphenyl-tetrazolium) assay. Briefly, cells were plated in 96-well plates at a density of 10,000 cells/100 μL of media containing 10% FBS, cell cycling-synchronized by the absence of FBS for 48 h, and then treated or not treated with PL for 24 and 48 h. One hundred (100) µl of a solution containing 90% medium and 10% 5 mg MTT/mL diluted in PBS was added and incubated for 4 h at 37°C. Next, 100 µl of SDS 10% solution was added to each well with the MTT solution and incubated in the dark in a plate shaker for 20 min. After 16 h, the absorbance was measured at 550 nm using a spectrofluorometer and plate luminometer (VarioScan Lux, Thermo Fisher).

### Cell cycle analysis

OSCC cells were synchronized for 48 h by serum starvation and released into medium containing 10% FBS. Cells were then treated with *PL* for 24 and 48 h; Then, they were collected, fixed in 70% ethanol for 30 min, treated with 10 μg/ml of RNAse (Sigma), and stained with 50 μg/ml of propidium iodide (Sigma). Cell cycle phase distribution was analyzed using a FACSCalibur flow cytometer (BD Biosciences, United States) equipped with an argon laser and ModFit LT software (Verity Software House, United States).

### Invasion assay

This assay was performed to examine the modulation of OSCC cells during invasion by treatment with PL metabolites. Briefly, the membranes of 6.5 nm transwell chambers with 8 µm pores (Corning, United States) were coated with 50ul of gelatinous matrix Miogel (2.4 mg/ml) with type I collagen (0.8 mg/ml) (Corning, Cat # 354236) (Salo et al., 2015)and incubated for 12–16 h. Then, the cells previously treated and non-treated with PL were plated in 200ul of DMEM/F12 medium without FBS, and subjected to the invasion assay according to Rodrigues AN et al. (2022) (Rodrigues et al., 2022). The invasion profile was assessed by measuring the absorbance of the cell dye at 650 nm using an Epoch plate reader (BioTek).

### Scratch wound healing assay

The cell migration ability was determined using a scratch wound healing assay. Cells (5 × 10^4^ cells/ml) were seeded in six well plates and incubated at 37°C until they were confluent. The monolayered cells were wounded by scratching with pipette tips and incubated further at 37°C for 24 and 48 h. Phase contrast images of the cells were captured at the time of scratching and afterward during the incubation, using a camera coupled to an inverted microscope (Nikon, Eclipse TS100) using Motic Image Plus 2.0 software. Cell migration into the wound area was calculated as the remaining space, using ImageJ v. 1.49(Grada et al., 2017).

### RT-qPCR

Total RNA was isolated using TRIzol reagent, according to the manufacturer**’**s protocol (Invitrogen, United States). Following DNase I treatment, 500 ng of total RNA per sample was used for reverse transcription using the commercial kit Luna OneStep qPCR (Biolabs) according to the manufacturer’s protocol. All qRT-PCR reactions were performed in triplicate using a quantitative thermal cycler 7500 Real-Time PCR System (Applied Biosystems). Human gene expression of markers relevant to migration and invasion (i.e., epithelial-mesenchymal transition markers *E-CAD (E-Cadherin), N-CAD (N-cadherin)*, and *VIM (vimentin*), and ECM remodeling markers (*MMP2* and *MMP9, and TIMP1 and TIMP2*) were determined in OSCC cell lines treated and not treated with PL metabolites using the relative quantification 2^−ΔΔCt^ method. The housekeeping gene, beta-actin (B-ACT), was used as a reference gene for data normalization. Gene expression analysis of mouse tongue tissue samples was carried out for the proliferation markers *Bcl2* and *Pcna,* and for the oral carcinogenesis-associated markers *E-cadherin (Cdh1), N-cadherin (Cdh2)*, *vimentin*, and *Twist*. Relative quantification was performed using the tongue tissue of sham animals as a reference control, and the mouse housekeeping *β-actin* gene was used for data normalization. At the end of the amplification, an additional dissociation step was included (45 cycles with a decrease of 1°C every 15 s, starting at 95°C) to generate a dissociation curve (melting curve) necessary to confirm the specificity of the amplified product. Sequences of the human and mouse primers used in this analysis are listed in Supplementary Table S1.

### Animals

Sixty-three Balb/c 4-week-old male mice were obtained from the Central Animal Facility, University of São Paulo (FMUSP), Brazil. The mice were maintained under standard conditions with a 12 h light/dark cycle, controlled temperature (24 ± 2°C), and free access to commercial chow and water *ad libitum*, according to the Ethics Committee in Animal Experimentation (protocol CEUA/FMJ 87/2017).

### OSCC chemical induction and PL extract treatment

The induction of oral carcinogenesis was optimized for our experiments as previously described(César Andrés Rivera Martínez, 2012; Bouaoud et al., 2021). Briefly, 4-nitroquinoline-1-oxide (4NQO) (Sigma-Aldrich, USA) was dissolved in water at 50 μg/mL and stored in a bottle protected by light at 22°C. Sixty experimental mice 6-week-old received 4NQO daily for 18 weeks, of which forty-five survived to the chemical induction. After the appearance of tongue lesions and verification of dysplasia development, induction was interrupted. Twenty-one mice were orally administered an aqueous solution of PL extract (Pharmacia, Brazil) at a final concentration of 10 mg/mL (+4NQO + PL), and other twenty-four mice received water only (+4NQO-PL), with an average volume of 5 mL of this solution per day per animal (Supplementary Figure S1). After 10, 17, and 20 weeks, the treated (+4NQO + PL) and non-treated (+4NQO-PL) animals were euthanized and their tongues were collected for microscopic analysis. The twenty-four animals of the untreated group received water *ad libitum* until the day of paired euthanized PL-treated mice. A group of three sham control mice not 4NQO-induced nor PL-treated was also included in the analysis.

### Histopathological analysis

Tongues from sham controls (n = 3) and mice treated with 4NQO (n = 45), followed by treatment with PL (n = 21/45) and without PL (n = 24/45), were fixed in 10% buffered formalin, embedded in paraffin, cut (5 µm sections), and stained with hematoxylin and eosin (H&E) (Pereira et al., 2007). Because some mice had more than one lesion per tongue, the total number of tissues/lesions per group analyzed was greater than the number of animals included in the study (Supplementary Figure S1). The animal data investigated for the histological and immunohistochemical analyses are summarized in Supplementary Table S2. Slides were evaluated by an examiner blinded to the group status who classified tumors using the following scoring system (Warnakulasuriya et al., 2021) (OMS Book):0, normal epithelial architecture; 1, mild dysplasia (changes limited to the basal third of the lining epithelium); 2, moderate (changes in two-thirds of the lining epithelium); 3, severe (more than two-thirds); 4, carcinoma *in situ* (full thickness of the lining epithelium, without invasion of connective tissue); and 5, invasive carcinoma (carcinomatous islands into connective tissue).

### Immunohistochemistry

Immunohistochemistry was performed using a rat anti-Ki-67 polyclonal antibody (Thermo Fisher Scientific, USA) diluted to 1:1000. Ki-67 expression was assessed using Aperio ScanScope CS (Aperio Technologies, United States). Briefly, 5 µm sections from twenty-seven FFPE tongue lesions of animals treated with PL, thirty-three lesions of the tongue of animals not treated with PL, and three normal tongue tissue specimens from controls (Sham) (Supplementary Figure S1) were deparaffinized, gradually rehydrated, and subjected to heat-mediated antigen retrieval in 10 mM citric acid (pH 6.0) in a pressure cooker. The sections were treated with 3% hydrogen peroxide to block endogenous peroxidase activity. Sections were incubated with the primary antibody (Ki-67) overnight, followed by LSAB (LSAB + System-HRP kit, Dako, United States) according to the manufacturer’s protocol. Detection was performed using 3.3′-diaminobenzidine tetrahydrochloride (Sigma-Aldrich, United States) containing a 0.01% H_2_O_2_ detection system. Control reactions were performed by omitting the primary antibodies. The slides were counterstained with Carazzi hematoxylin. The slides were scanned into high-resolution images that were analyzed using the pixel count V9 algorithm (Aperio Technologies, United States). Tongue tissue immunoexpression of Ki-67 was analyzed semi-quantitatively using the following criteria: the percentage of positivity was graded from 0 to 4 (0: no staining; 1:1%–25% staining; 2:26%–45% staining; 3:46%–75% staining; 4:76%–100% staining), and tongue lesion samples were classified as presenting Ki-67 lower (1–2) or higher (3–4) expression (Supplementary Table S2).

### Statistical analysis

All *in vitro* assays were performed at least three times, and data were analyzed using one- or two-way analysis of variance with repeated measures (RM-ANOVA) with *post hoc* comparisons based on Tukey’s or Dunnett’s multiple comparison test. Gene expression analysis for markers verified among different tongue tissues was performed and compared using the Kruskal–Wallis test with Dunn’s *post hoc* test. These analyses were performed using the GraphPad Prism software version 6.0. For all statistical analyses, the significance level was set to 5% (*p* ≤ 0.05).

## Results

### PL treatment led to a decrease in viability and proliferation and promoted apoptosis in OSCC cells

**FIGURE 1:**
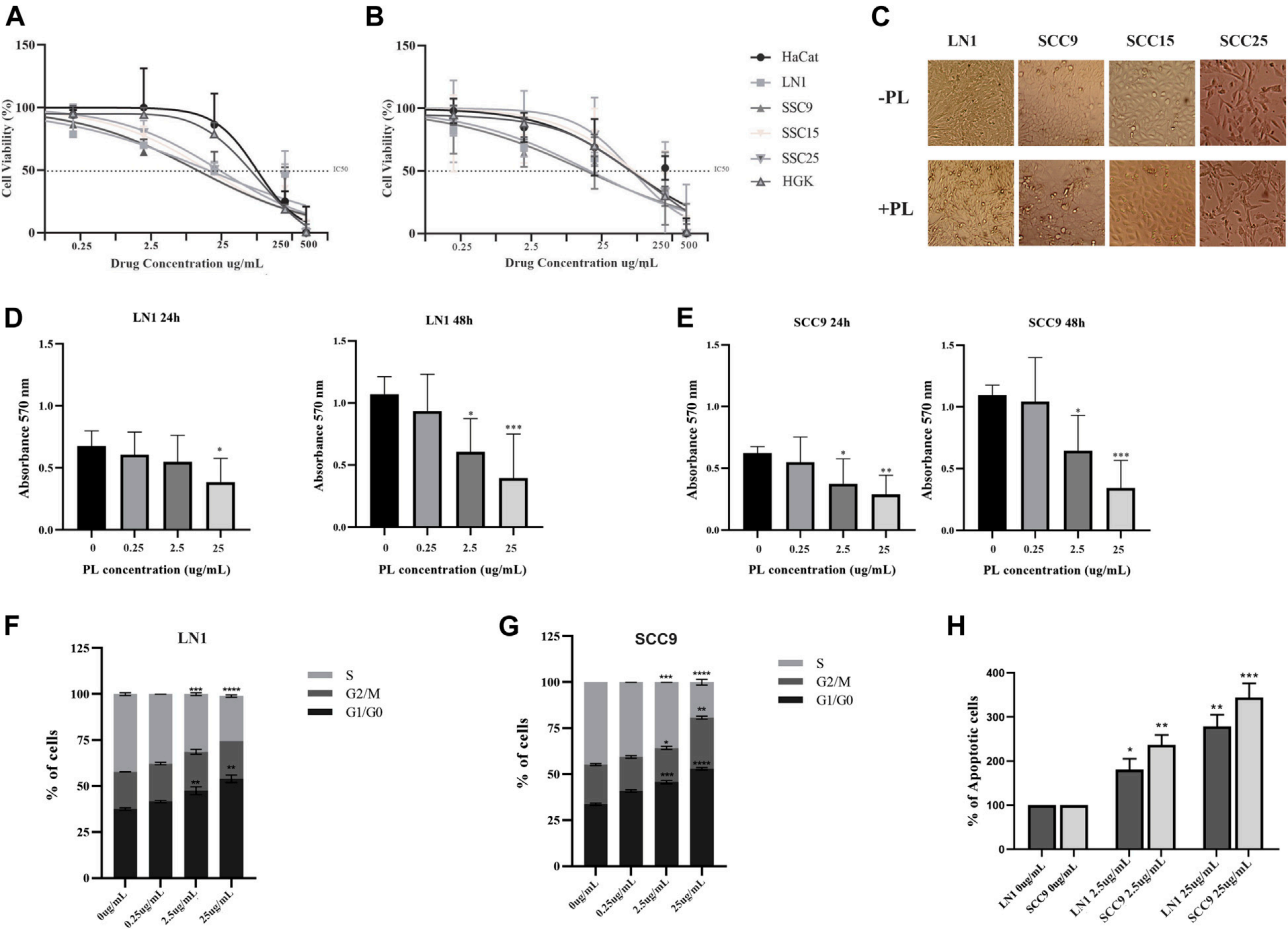
PL inhibits proliferation and enhances apoptosis of OSCC cells. Cells were subjected to IC50 test **(A-B)**, proliferation assay **(C-E)**, DNA content cell cycle analysis **(F-G)**, and apoptosis **(H)** assays. The cytotoxicity test showed an IC50 of Non-transformed (HaCat and HGK) and OSCC cell lines (SCC9, SCC15, SCC25, LN1) after 24 h **(A)**, and after 48 h **(B)** treatments with PL. Representative images showing PL treatment effect on OSCC cells morphology **(C)**. Cell Proliferation assay of LN1 **(D)** and SSC9 **(E)** cell lines incubated with selected PL concentrations, compared to non-treated cells. Quantification of cell cycle analysis was performed by flow cytometry after staining with propidium iodide in the LN1 **(F)** and SSC9 **(G)** cell lines treated with selected PL concentrations and non-treated cells. PL treatment induced arrest of the OSCCs cell cycle in the G1 phase, decreasing proliferation. **(H)** Flow cytometric analysis of apoptosis showed a remarkable increase in the number of apoptotic cells for cells treated with PL. Plots compose an experimental triplicate analysis and were statistically imposed using ANOVA followed by Tukey’s test, where *p < 0.05, **p < 0.01, ***p < 0.001, ****p < 0,0001.

To gain insight into the role of PL metabolites in OSCC progression, the cell lines SSC-9, SCC-15, SCC-25, and LN1 were treated with PL metabolites at concentrations of 0.25, 2.5, 25, 250, and 500 ug/ml. The effective concentrations of PL metabolites required to kill 50% (IC 50) of each oral tumor cell are shown in Figures 1A,B. The two OSCC cell lines that responded best to PL treatment were SCC-9 and LN1, both with highly proliferative phenotypes. The IC50 values for LN1 were approximately 20 and 25 ug/ml after 24 h (Figure 1A) and 48 h (Figure 1B), respectively. In contrast, SCC-9 had inhibitory concentration of 15 and 20 ug/ml during treatment for 24 h (Figure 1A) and 48 h (Figure 1B), respectively. Although similar IC was found for both SCC-15 and SCC-25 cell lines after 24 h of treatment with PL (−25ug/ml), at 48 h treatment a much higher IC dose was determined (−115ug/ml) (Figures 1A,B). Both non-transformed cells, HaCat and HGK, showed similar and not significant cytotoxic effect after treatment with PL. We also determined the sublethal IC at 25% of dead cells, which included doses of PL between 2.2 and 25 ug/ml for the OSCC cell lines used in this analysis for the majority of OSCC-responsive cells. The effect of PL on morphology of OSCC cells is represented in Figure 1C, showing apoptotic morphology for cells under sublethal concentrations treatment.

To gain insights into the influence of PL on oral carcinogenesis, *in vitro* experiments were carried out using the three PL concentrations determined, including sublethal treatments (25% of dead cells) and a lethal treatment (at least 50% of dead cells), which ranged from concentrations of 0.25–25 ug/ml. The concentrations for PL treatment were chosen based on the 2 cell lines that responded better to the IC50 assay, with which we carried out all the following *in vitro* experiments with SCC-9 and LN1 cells. Although both cells treated with PL showed affected, it was possible to observe that PL concentrations drastically impaired the proliferation of the LN1 at 24 h (25 ug/mL, *p* < 0.05) and 48 h (2.5 ug/mL, *p* < 0.05; 25 ug/mL, *p* < 0.001) (Figure 1D). In addition, proliferation of SCC-9 cells was affected by both doses of PL at 2.5 ug/mL (*p* < 0.05) and 25 ug/mL (*p* < 0.01), after for 24 h incubation (Figure 1E). Accordingly, both doses of PL, at 2.5–25 ug/ml, enhanced the number of cells at the G0–G1 phase for both LN1 (*p* < 0,0001) (Figure 1F) and SCC-9 cells (*p* < 0,0001) (Figure 1G). An important reduction in the S phase in both cell types (*p* < 0,0001) was observed compared to that in the non-treated controls (Figures 1F,G). In addition, treatment with PL metabolites at IC50 (*p* < 0.01) and higher dose (*p* < 0.001) concentrations showed a significant increase in apoptotic SCC-9 (*p* < 0.05) and LN1 (*p* < 0.001) oral cells, compared with non-treated PL cells (Figure 1H).

### PL treatment inhibits the migration and invasion of tumor cells and the EMT process

**FIGURE 2:**
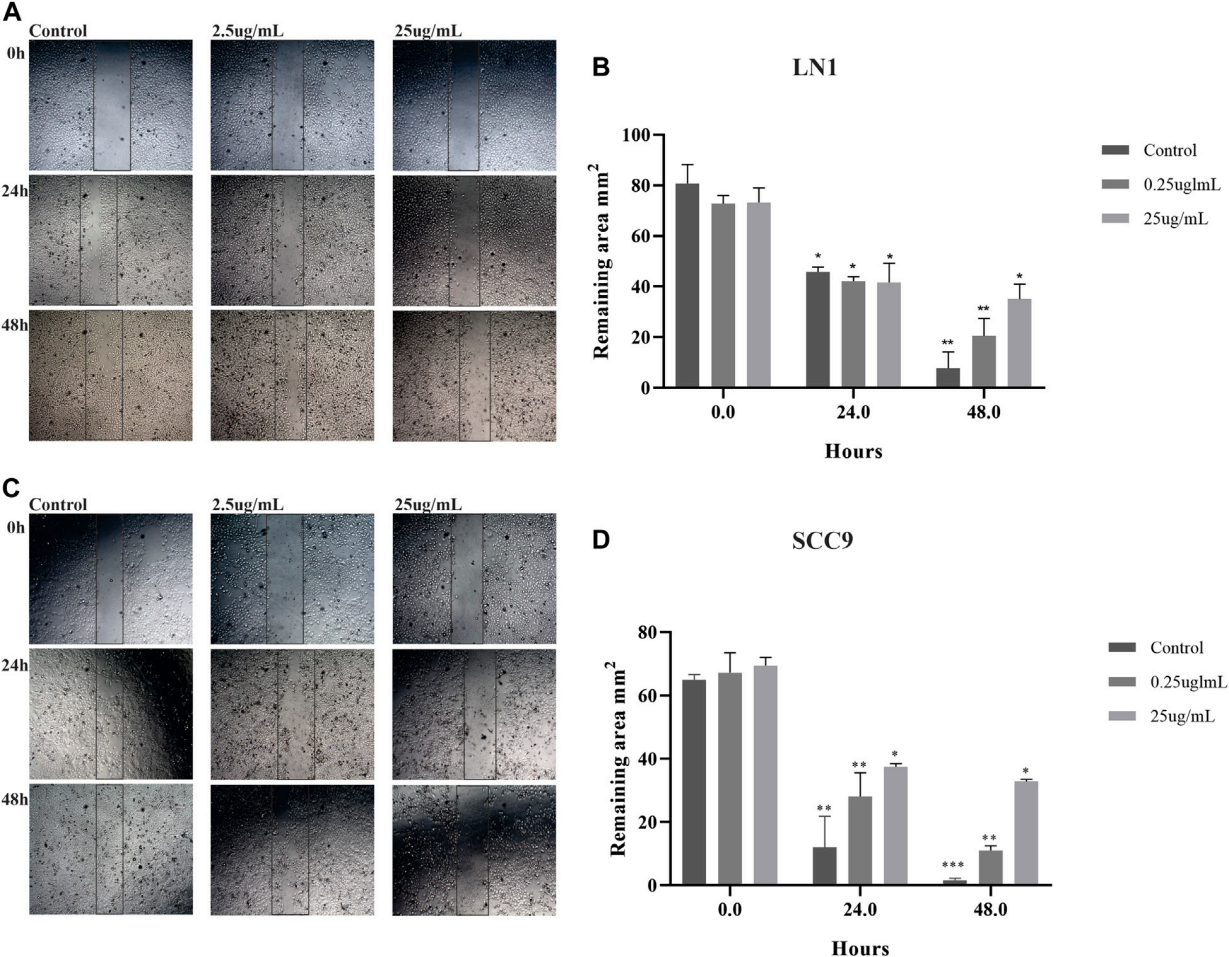
PL treatment decreases the migration of OSCC cell lines. Photomicrographs of cell lines were taken at 0, 24, and 48 h after wounding (40X). The average width of the lacunae was measured. Migration of SCC-9 cells treated with PL was significantly decreased, in comparison with non-treated cells for both 24 and 48 h of treatment **(A)**. LN1 cells treated with PL migrated significantly less for 48 h incubation **(B)**. Migration analysis based on the scratch wound assay showed that cells treated with PL closed the scratch wound significantly slower than the cells not treated (control). The graphs compile experimental triplicate quantification analyses of the remaining area, and the results were obtained by Two-way ANOVA followed by Tukey assay, where *p < 0.05 , **p < 0.01 , and ***p < 0.001.

**FIGURE 3:**
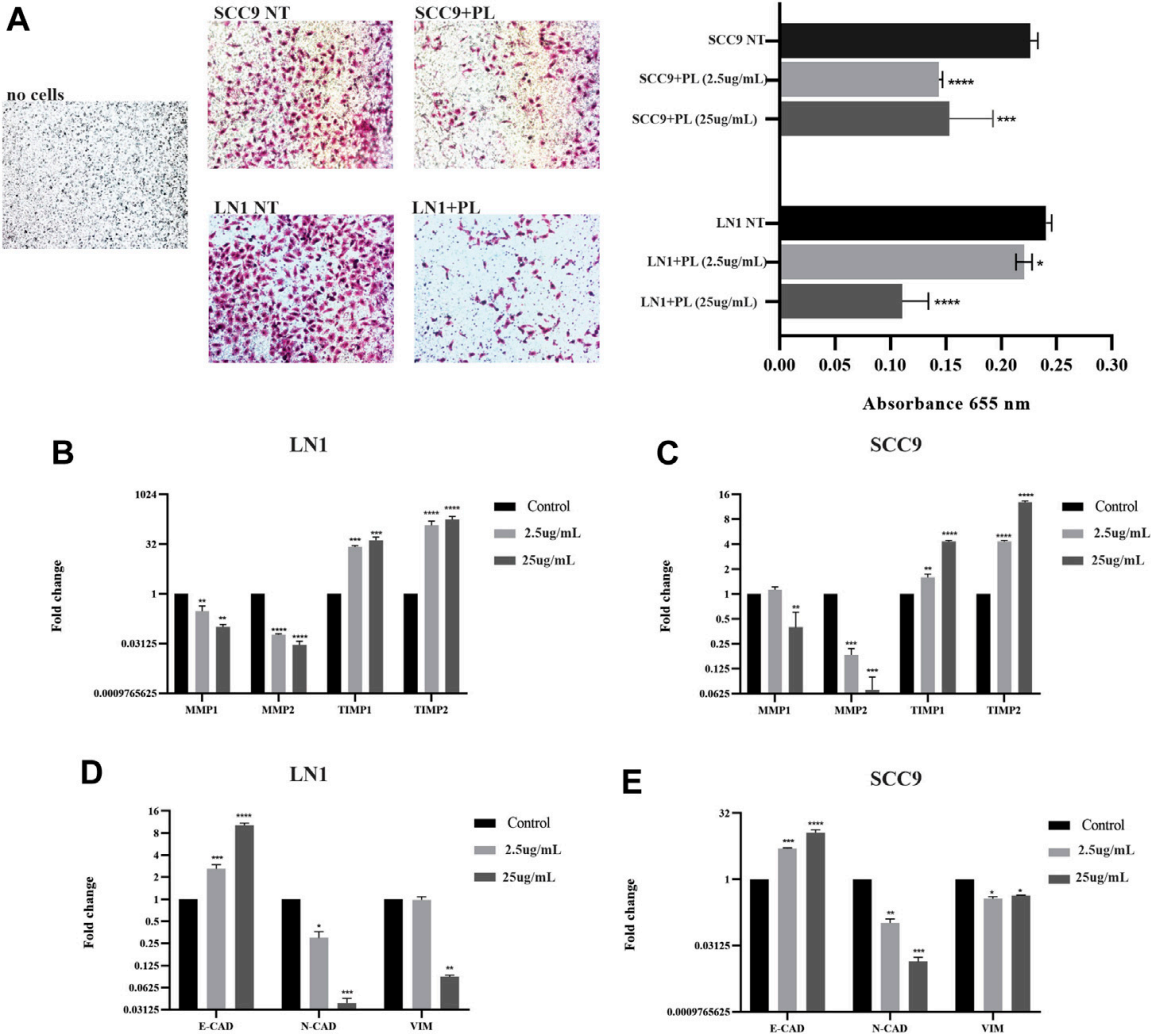
PL suppresses invasion and inhibits ECM markers and EMT properties in OSCC. Invasion of SCC-9 and LN1 OSCC cells was significantly inhibited by treatments with PL **(A)**. The ECM markers, MMP1 and MMP2, were significantly downregulated, and their regulators TIMP1 and TIPM2, were significantly upregulated after the treatment of OSCC cell lines with PL **(B-C)**. PL treatment significantly induced the expression of E-CAD while reducing N-CAD and VIM expression in both cell lines **(D-E)**. The graphs compile experimental triplicate analyses, and the results were obtained by ANOVA followed by Tukey assay, where *p < 0.05, **p < 0.01, ***p < 0.001, ****p < 0.0001.

Migration and invasion assays were performed using cells treated with sublethal concentrations up to 25 ug/ml. Although PL significantly negatively modulated the migration potential of LN1 cells at 2.5 ug/ml (*p* < 0.01) and 25 ug/ml (*p* < 0.001), it did so after a prolonged incubation (Figure 2A). The scratch wound migration assay of the SCC-9 cells treated with PL, at 2.5 ug/mL (*p* < 0.01) and 25 ug/mL (*p* < 0.001), revealed significantly lower migration, particularly with the higher concentration treatment, for both periods of incubation with PL, compared to non-treated control (Figure 2B). In the invasion assay, we observed that LN1 cells had a significant reduction in invasiveness of approximately 60% when treated at 2.5 ug/mL of PL (*p* < 0,0001), and about 15% reduction when treated at 25 ug/ml of PL, compared to the non-treated controls (Figure 3A). SCC-9 cells showed a decrease in invasion of approximately 45% (*p* < 0.001) at 2.5 ug/ml, and 40% (*p* < 0,0001) at 25 ug/ml μg/mL PL, compared to non-treated cells (Figure 3A). To explore the molecular outcome of PL modulation in these OSCC cell lines, we verified the gene expression of the canonical EMT-associated pathways. PL-treated (2.5 and 25 ug/mL) cells were quantified by RT-qPCR to examine its mRNA levels modulation of E-CAD, VIM, N-CAD, MMP1, MMP2, TIMP1, and TIMP2 genes compared to non-treated cells.

LN1-PL treated cells, showed significantly lower MMP-1 (*p* < 0.001) and MMP-2 (*p* < 0,0001) expression levels, and higher TIMP-1 (*p* < 0.001) and TIMP-2 (*p* < 0,0001) transcripts at both concentrations of PL, compared to non-treated cells (Figure 3B). The levels of MMP-1 (*p* < 0.01) and MMP-2 (*p* < 0.001) were also significantly downregulated, and TIMP1 (*p* < 0.001) and TIMP2 (*p* < 0.0001) were highly expressed in the SCC-9 PL-treated cells compared to those in the controls (Figure 3C). In addition, OSCC cells treated with PL acquired EMT-reversed properties (mesenchymal-epithelial transition), such as lower expression levels of the mesenchymal markers N-CAD (*p* < 0.001) and VIM (LN1, *p* < 0.01; SCC-9, *p* < 0.05) and higher expression of the epithelial marker E-CAD (*p* < 0.0001), particularly when treated with 25 μg/mL PL (Figures 3D,E).

### Immuno-inflammatory tumor response to macrophage polarization associated with OSCC cells treated with PL

**FIGURE 4:**
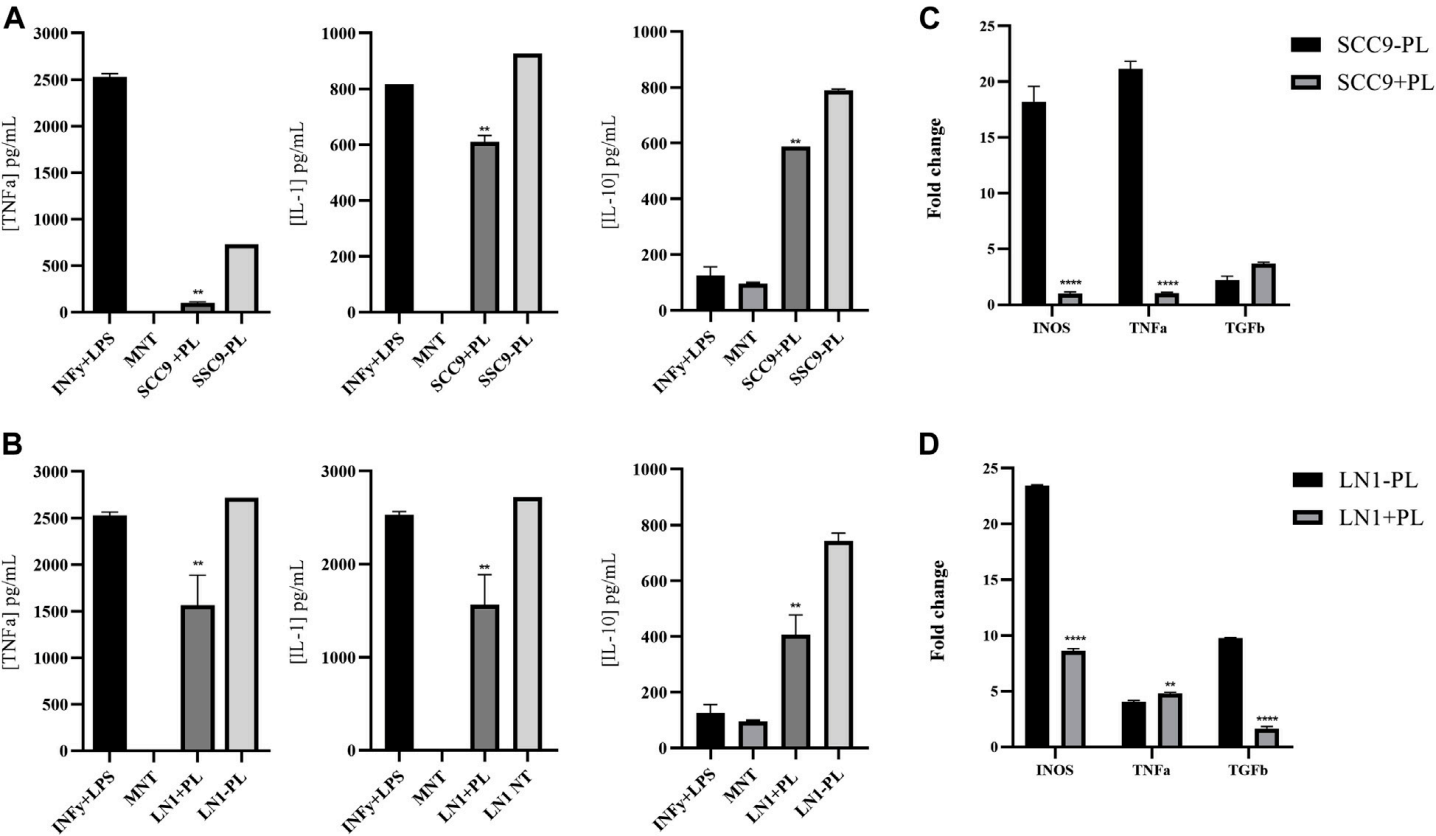
Immuno-inflammatory tumor response associated with OSCC cells treated with PL. Showing quantification of the cytokines IL-1, IL-10 and TNF-alfa produced by macrophages conditioned with tumoral media treated with PL **(A-B)**. Incubation with both SCC9 **(A)** and LN1 **(B)** cells treated with PL significantly decreased the secretion of TNF-alfa (SCC9, *p* < 0.001; LN1, *p* < 0.01) and IL-1 (SCC9, *p* < 0.01; LN1, *p* < 0.01). Gene expression analysis of Macrophages incubated with SCC-9 tumoral media treated with PL showed significant upregulation of TGF-beta, and downregulation of TNF-alfa and iNOS, compared to tumoral media not treated with PL **(C)**. Expression analysis of Macrophages incubated with LN1 tumoral media treated with PL showing significant downregulation of TGF-beta and iNOS **(D)**. The graphs compile experimental duplicate analyses, and the results were obtained by ANOVA followed by Tukey assay, where **p < 0.01, and ****p < 0,0001.

Because inflammation is an important mediator of carcinogenesis associated with EMT induction, we investigated the role of PL treatment in OSCC cell lines during macrophage polarization. To this end, we measured the cytokines IL-1, IL-10, and TNF-α produced by these tumor-conditioned macrophages. Incubation of macrophages with both LN1 and SCC-9 cells treated with 2.5ug/ml of PL significantly decreased its secretion of TNF-alfa (*p* < 0.01) and IL-1 (*p* < 0.01) (Figures 4A,B). Interestingly, the expression of IL-10, an immunosuppressive cytokine, was also significantly decreased (*p* < 0.01) in the media of macrophages incubated with both SCC-9 and LN1 tumoral-conditioned media containing PL (Figures 4A,B). In addition, the expression profile of the tumor-immunoregulatory genes *TNF-*α, *TGF-β*, *IL-10*, and *iNOS* was determined in macrophages conditioned with OSCCs tumoral media previously treated with PL, in comparison with non-treated cells (Figures 4C,D). Our results demonstrated that macrophages incubated with SCC-9 conditioned tumoral media containing PL showed significant upregulation of TGF-β (*p* < 0.05) and significant downregulation of TNF-α (*p* < 0.0001) and iNOS (*p* < 0.0001), compared to conditioned tumoral media without PL (Figure 4C). On the other hand, incubation of LN1 conditioned tumoral media with PL showed significant downregulation of TGF-β (*p* < 0.0001) and iNOS (*p* < 0.001), but no significant difference in TNF-α expression was found, compared to LN1 conditioned tumoral media without PL (Figure 4D).

### PL treatment affects OSCC development *in vivo*

**FIGURE 5:**
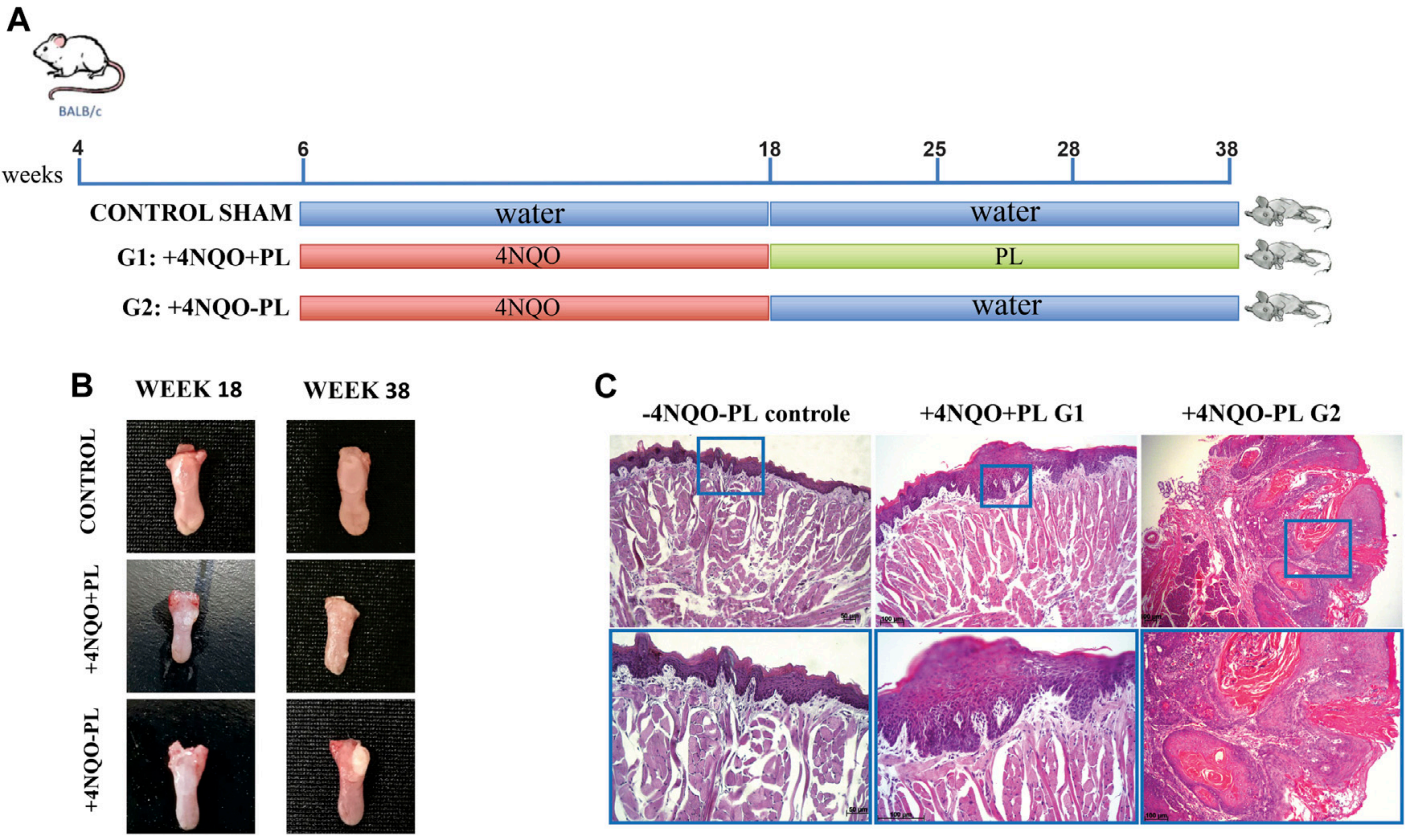
Representative photomicrographs of histopathological analysis of the tongue epithelium after carcinogenic induction and PL treatment. **(A)** Timeline of BALB/c mice chemical carcinogenesis induction by 4-NQO. **(B)** Macroscopy: representative tongue of control mice, which did not receive 4NQO induction, and without tongue lesion; the tongue of mice induced with 4NQO and treated with PL (+4NAO + PL); and tongue of mice induced with 4NQO and not treated with PL (+4NQO-PL). **(C)** Histological microscopy: -4NQO-PL, normal mouse epithelium that did not receive chemical induction (control); +4NQO + PL, stratified epithelium showing moderate dysplasia, representative of animals 4NQO-induced that were treated with PL; and +4NQO-PL, representative well-differentiated SCC of the majority of mice that were 4NQO-induced and were not treated with PL, showing intense epithelial dysplasia involving the entire thickness of the epithelium and areas of loss of basement membrane integrity. Upper panel: photomicrographs at × 100 magnification; lower panel: photomicrograph at × 200 magnification.

Sixty BALB/c mice were subjected to 4-NQO chemical induction; however, during this process, there was a loss of 15 mice (approximately 25%), which has been commonly reported in the literature for oral cancer chemical induction(César Andrés Rivera Martínez, 2012). From the remaining 45 animals, we found that the experimental groups treated with 4-NQO had visible lesions. Upon gross examination, the visible lesions appeared as single or multiple masses of various sizes, as is typically observed in cancers of the oral cavity. A histological examination confirmed the presence of dysplastic lesions. Because there was no significant difference in PL treatment among the different periods, the subgroups treated for 10, 17, and 20 weeks with PL were pooled for the analyses, and the data were generated considering two large groups: PL-treated (n = 21) and non-treated (n = 24) (Figure 5A). The average consumption of the PL treatment solution ranged from 18 to 40 mL per week, with an average of 22 mL per week/animal (+4NQO + PL). Water consumption without treatment with PL (+4NQO-PL) varied from 19 to 40 mL per week, with an average of 23.25 mL per week/animal. Hence, the PL solution was palatable as the animals consumed very similar values.

All longitudinal sections of the tongue tissue samples were subjected to histological examination by a trained pathologist who was blinded to sample identities. Microscopic evaluation of the majority of the lesions in animals treated with 4NQO demonstrated the development of multifocal lesions, particularly in the +4NQO-PL group. In this group, 33 lesions, ranging from mild dysplastic to invasive-differentiated bulk OSCCs, were observed (Figure 5B), with a predominance of severe lesions and TSCCs (72.7%), followed by moderate (15.2%) and mild (12.1%) (Supplementary Figure S1). In the +4NQO + PL group, the lesions (n = 27) appeared macroscopically smaller (Figure 5B). In this group, mild to moderate injuries (63%) predominated, although severe dysplasia (15%) and well-differentiated TSCCs (22%) were also observed (Supplementary Figure S1). Notably, three mice did not have any dysplastic lesions and were further excluded from the analyses. Microscopically, papilloma, characterized by epithelial hyperplasia with the formation of hyper-orthokeratinized fingerlike projections, was also observed in the treated animals (Figure 5C). However, in the group of samples evaluated from animals not treated with PL, a significantly higher incidence of moderate-to-poorly differentiated TSCC was observed, with an epithelium characterized by the abundant production of keratin by neoplastic epithelial cells. There was also intense epithelial dysplasia involving the entire thickness of the epithelium and areas of loss of integrity of the basement membrane in addition to an inflammatory infiltrate in the underlying connective tissue (Figure 5C). In contrast, histopathological analyses of the control animals (Sham) demonstrated preservation of the integrity of the basal lamina, revealing the presence of orthokeratinized stratified squamous epithelium with few cell layers (Figure 5C).

**FIGURE 6:**
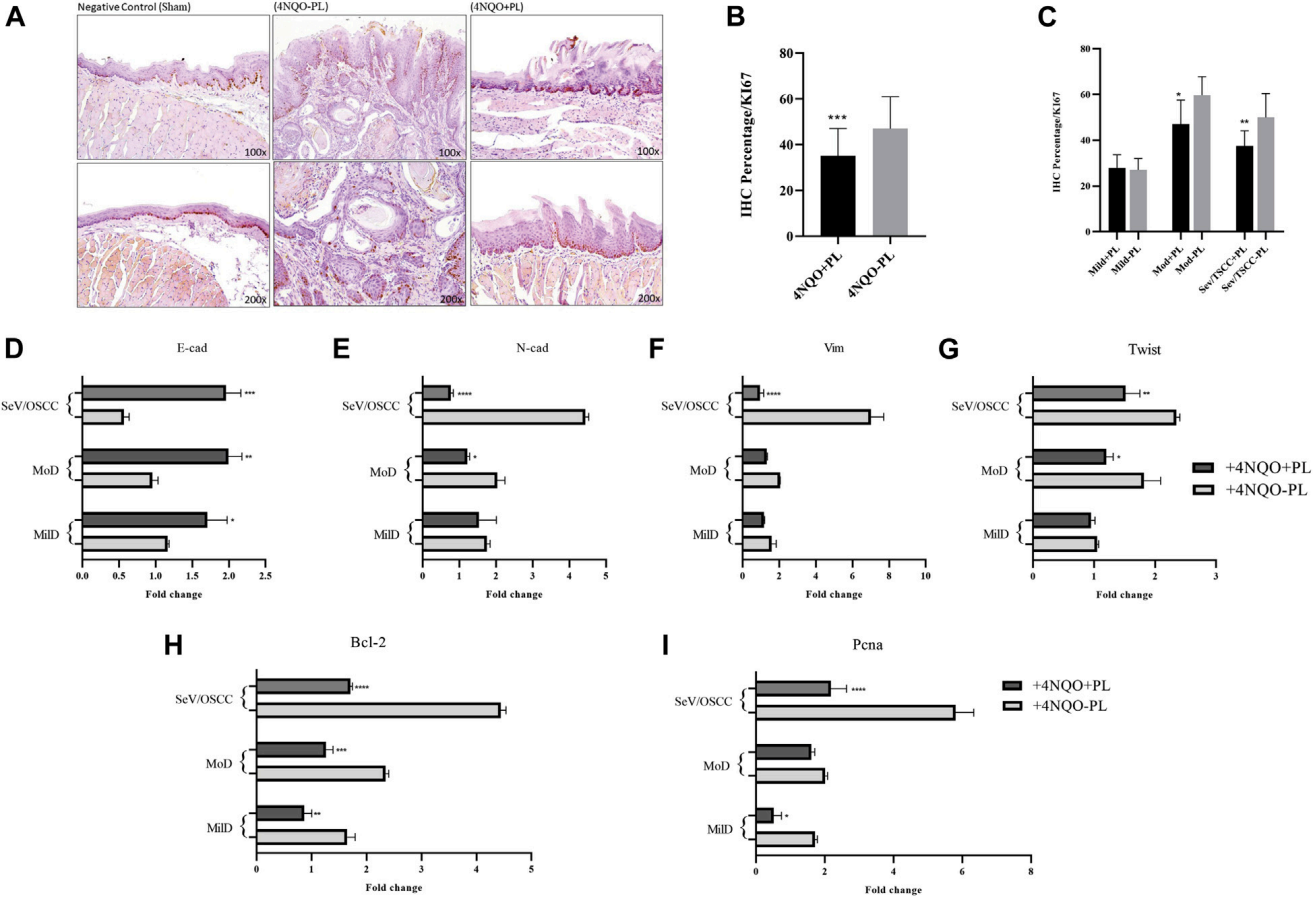
Representative image of Ki-67 IHC analysis of histological sections of the tongue of mice 4NQO-induced and treated or not with PL. **(A)** Representative IHC of the tongue tissue of control mice without 4NQO-induction (Sham), showing Ki-67 staining in the epithelial basal layer; (+4NQO-PL) Cells exhibiting positive immunoreactivity for Ki-67 in Tongue Squamous cell carcinoma *in situ* (mice not treated with PL), exhibiting staining and distribution in the basal, suprabasal and spinous layers; (+4NQO + PL) Moderate to weak staining of Ki-67 in area of epithelial dysplasia of mice treated with PL. Upper panel: ×100 magnification; lower panel: ×200 magnification. **(B)** Overall quantification of Ki-67 showed a significantly lower expression of this marker in the group of mice treated with PL. **(C)** Distribution of Ki-67 staining among tongue lesions of animals treated and not treated with PL; showing significantly lower expression of Ki-67 in moderate dysplasia and SCC of PL-treated mice. **(D–G)** Gene expression analysis of EMT markers E-cadherin, N-Cadherin, Vimentin, and transcription factor Twist 1, in oral dysplastic and Tongue SCC tissues of mice treated with PL, in comparison with not-treated. **(H–I)** Quantification mRNA expression of genes associated with proliferation, Bcl2 and Pcna, in oral dysplastic and SCC tissues of mice treated with PL, in comparison with not-treated. The graphs compile experimental duplicate analyses, and the results were obtained by ANOVA followed by Tukey assay, where *p < 0.05, **p < 0.01, ***p < 0.001, ****p < 0.0001.

Ki-67 immunostaining was performed in 27 tongue lesion tissues of the tongue tissues of treated animals and 33 lesions of animals not treated with PL (Supplementary Table S2). This analysis was first performed by comparing the overall Ki-67 expression in the lesions of the tongues of PL-treated and non-treated animals. Furthermore, we subdivided these lesions according to their histological classification into mild, moderate, and severe + SCC in both groups. Our results showed significantly higher expression of Ki-67 (*p* < 0.001) in the tongue tissues of non-PL-treated animals than in the PL-treated group (Figures 6A,B). In particular, moderate and severe dysplasia and TSCCs had significantly lower expression (*p* < 0.05) of this proliferation marker in animals treated with PL (+4NQO + PL) than in the same category of lesions in the +4NQO-PL group (Figure 6C). Notably, the expression of Ki-67 was restricted to the basal layers in the normal epithelium of Sham mice, whereas Ki-67 positive cells in tongue epithelial dysplasia were located in the basal, suprabasal, and spinous layers (Figure 6A). The distribution of higher Ki-67-expressing cells was significantly more abundant in the lesions of mice not treated with PL, and the predominance of lower Ki-67 expression was observed within the dysplastic lesions of mice treated with PL (Supplementary Table S3).

### Effect of PL on cell proliferation and epithelial-mesenchymal transition markers in tongue epithelial lesions

To explore the molecular outcome of PL modulation *in vivo*, we verified the gene expression of the canonical EMT- and proliferation-associated pathways. The mRNA levels of tongue lesions in PL-treated and untreated mice were quantified using RT-qPCR. In agreement with our *in vitro* results, TSCC and severe dysplastic epithelial cells from PL-treated mice acquired EMT-reversed properties (mesenchymal-epithelial transition), showing significantly lower expression of the mesenchymal markers vimentin and N-cadherin (*p* < 0.0001) and higher expression of the epithelial marker E-cadherin (*p* < 0.001) (Figures 6D–F). The lesions in mice treated with PL also showed a significant decrease in the expression of the EMT transcription factor, Twist1 (*p* < 0.01) (Figure 6G). Moderate dysplasia in PL-treated mice had significantly higher E-cadherin (*p* < 0.01) and lower N-cadherin (*p* < 0.05) and Twist1 (*p* < 0.05) expression than non-treated animals (Figures 6D,E,G). Regarding mild dysplasia, only E-cadherin was significantly upregulated (*p* < 0.05) following PL treatment in mice (Figure 6D).

In addition, decreased expression of *Bcl2* was observed in all dysplastic lesions and TSCCs treated with PL compared to that in non-treated mice (Figure 6H). PL treatment also significantly diminished *Pcna* expression in severe dysplasia + TSCC (*p* < 0.0001) and mild dysplasia (*p* < 0.05) compared to the same group of lesions in non-treated mice (Figure 6I).

## Discussion

Natural products serve as vital resources for cancer therapy (*e.g*., *vinca* alkaloids, camptothecin, and paclitaxel) and are sources of novel drugs. Drugs developed to specifically target tumor-related proteins represent the basis of precision medicine (Hientz et al., 2017). Thus, natural products from plants can serve as excellent resources for targeted therapies. Phytochemicals and herbal mixtures act multispecifically, *that is,* they attack multiple targets simultaneously. *Polypodium leucotomos* (PL) was chosen as a candidate antitumor agent in this study because of its multiple advantages. PL is believed to exert clinical benefits through its antioxidant, immunomodulatory, anti-inflammatory, and antimutagenic properties (Cyr and Richer, 2021). PL also has an excellent safety and tolerability profile(Parrado et al., 2020), and is a natural mixture of phytochemicals with powerful antioxidant properties (Gonzalez et al., 2010). Another striking property of PL is that it exerts its effects when administered orally.

In the present study, PL was used *in vitro* to treat OSCC cell lines, and our results showed decreased tumor cell viability, proliferation, migration, and invasion and enhanced cell apoptosis. Additionally, *in vivo* evaluation of PL treatment demonstrated its potential to prevent 4NQO-induced oral carcinogenesis in mouse tongues. PL also impaired the expression of proliferation, invasion, and metastasis genes, which are associated with ECM and EMT-associated, both *in vitro* and *in vivo*.

Upon great variability during the assays initially carried out with the pure PL plant extract treatment on oral cancer cells, and revising the literature on the phenolic compounds present in PL, we determined that the PL extract needed to go through a stage of metabolization for the formation of its active metabolites, which were then able to perform proper action in some cell lines (Gombau et al., 2006). This makes sense as herbal medicines have oral and topical routes of administration. The latter has been particularly used, especially in sunscreens, as a treatment for conditions associated with inflammation and skin conditions that appear to be associated with photodamage caused by UV radiation (Rodríguez-Yanes et al., 2014; Bhatia, 2015). When considering the oral administration route of PL, which has already been used in human clinical studies for skin conditions (Gombau et al., 2006; Berman et al., 2016; del Rosso, 2016), there is a need to metabolize this extract for the release of metabolites that can actively behave in other specific molecular pathways, mainly associated with antioxidant and antitumor cellular processes. Our *in vivo* data, whereby the herbal medicine extract was administered orally and, therefore, metabolized in the hepatocytes of these animals, showed surprising results regarding the inhibitory effect on the progression of OSCC in animals treated with PL compared to the untreated ones.

Gombau et al. (2006) (Gombau et al., 2006) also demonstrated that these compounds are easily absorbed after oral administration, which is decisive in choosing the treatment method for *in vivo* experiments. In addition, the same study demonstrated that compounds, such as hydroxycinnamic and benzoic acids, are not accumulated by enterocytes and are better absorbed at lower concentrations than at higher concentrations. This allowed us to infer that the transport of these phenolic derivatives in the PL occurred through a saturation-sensitive system. It is also known that, despite the rapid absorption of the phenolic derivatives in question, they are metabolized slowly by the body, involving different enzymatic systems, such as cytochrome P-450 and NADPH, normally leading to products with more polar characteristics and active metabolites (Gombau et al., 2006).

After determining the effective doses of PL metabolites to kill at least 50% of oral tumor cell lines, we treated OSCC cells to better understand the role of PL as an anti-tumor agent. Our study confirmed that the inhibition of proliferation resulted from the treatment of OSCC cell lines with PL at a concentration of 2.5 ug/ml or higher, as well as an increase in the number of apoptotic cells. We also demonstrated that PL has a dose-dependent effect, so tumor lines treated with higher concentrations of PL (25 ug/ml), and for a prolonged period (48 h), had a greater effect on the death of these tumor cells. So far, very few studies have evaluated the influence of PL on cancer cells(Parrado et al., 2020), including the fact that PL treatment decreased UV-induced epidermal cell proliferation and enhanced apoptosis through p53 increased expression(Rodríguez-Yanes et al., 2012).

We then explored the association between migration and invasion processes and PL treatment in oral cancer. We also demonstrated that PL metabolites can regulate the migration and invasion status of OSCC cells, which may explain why PL treatment significantly inhibited oral cancer progression, as demonstrated in our *in vivo* results. Notably, this is the first study to show a link between metastasis and tumor progression after PL treatment. Our results showed that SCC-9 cell lines incubated with PL metabolites at an initial concentration of 2.5 ug/ml and higher significantly reduced the ability of cancer cells to migrate. On the other hand, the LN1 cells seemed more resistant to PL treatment at 2.5 ug/ml dosage towards the migration process, and significantly decreased migration of these cells at a final concentration of 25 ug/ml of PL for prolonged incubation. In addition, a significant reduction in invasion was observed in SCC9 and LN1 cells following PL treatment. Interestingly, the invasion potential of OSCCs cells was more pronounced at lower concentrations of PL. This may be explained by the fact that a higher concentration of phytochemicals with fewer viable cells remaining is likely to become clones that are less responsive to higher doses of treatment. It is well known that resistance to chemotherapy drugs can be also acquired. In the case of acquired resistance, it is proposed that during the treatment of the tumor, which is initially sensitive to the drug, mutations may occur in some tumor cells, which would activate compensatory signaling pathways, allowing these cells to no longer respond to the treatment, which would confer a proliferative advantage concerning the total tumor mass (Holohan et al., 2013).

The hallmarks of cancer include cell growth and metastasis, which are facilitated by ECM degradation and EMT processes. The ECM, which gives the tissue its structural integrity, is remodeled in cancer *via* increased expression and activity of matrix metalloproteinases (MMPs) and inhibition of tissue inhibitors of matrix metalloproteinases (TIMPs). In the present study, the modulation of migration and invasion processes in OSCC cell lines treated with PL resulted in modulation of the expression of ECM and EMT markers. It was demonstrated that by treating both OSCC cell lines with PL, we observed a significant decrease in MMP-1 and MMP-2, and an increase in TIMP-1 and TIMP-2, compared to untreated cells. In agreement with our results, a study in human melanoma cells demonstrated that PL can beneficially modulate cancer cell growth and ECM remodeling markers by antagonizing the stimulation of MMP-1, TGF-β, and heat-shock proteins and enhancing the expression of TIMP-1 while maintaining growth inhibition(Philips et al., 2009b).

For the first time, we demonstrated that PL could also modulate EMT markers in cancer cell lines. Both OSCC cell lines treated with PL showed enhanced expression of the epithelial marker, E-CAD, and diminished expression of the mesenchymal marker, N-CAD. VIM gene expression was significantly downregulated in SCC-9 cells at both concentrations, but it only resulted in significantly decreased expression at a sublethal concentration of PL treatment in LN1 cells.

Epithelial-mesenchymal transition is a dynamic process in which the migratory capacity and invasiveness of epithelial cells are enhanced by the loss of intercellular adhesion and polarity (Vallina et al., 2021). It is characterized by the endowment of cells with a mesenchymal phenotype as they lose their epithelial features. Classic EMT markers include cadherin, catenin, and vimentin. Cadherins are a family of transmembrane proteins that, together with catenins, participate in cell adhesion, and vimentin is the main component of the cytoskeleton of mesenchymal cells(Vallina et al., 2021). In OSCC, E-CAD (which promotes adhesion of epithelial cells) levels decrease, while levels of N-CAD and VIM increase (which favors cell invasion and migration) (Zhang et al., 2021). Similarly, decreased E-CAD expression and increased N-CAD and VIM expression are associated with distant metastasis and decreased survival in patients with OSCC. Our results suggest that PL treatment can inhibit EMT in OSCC by upregulating E-CAD and downregulating N-CAD and VIM, as shown by our *in vitro* and *in vivo* data.

M1 macrophages are traditionally regarded as antitumor, whereas M2-polarized macrophages, commonly termed tumor-associated macrophages (TAMs), contribute to many pro-tumorigenic outcomes in cancer through angiogenic and lymphangiogenic regulation, immune suppression, hypoxia induction, tumor cell proliferation, and metastasis. The TME can influence macrophage recruitment and polarization, giving way to these pro-tumorigenic outcomes; (Boutilier and Elsawa, 2021); thus, investigating TME-induced macrophage polarization is critical for further understanding TAM-related pro-tumor outcomes and the potential development of new therapeutic approaches.

In the present study, we also investigated the expression profile of tumor immunoregulatory genes TNF-α, TGF-β, IL-10, and iNOS in macrophages conditioned with OSCC tumoral media treated with PL during polarization. We demonstrated that macrophages incubated with SCC-9 tumoral media treated with PL showed significant upregulation of TGF-β and downregulation of TNF-α and iNOS compared to tumoral-conditioned media not treated with PL. On the other hand, incubation with LN1 tumoral media treated with PL showed significant downregulation of TGF-beta and iNOS, but not of TNF-alpha, compared to LN1 untreated conditioned media. In addition, we measured the cytokines IL-1, IL-10, and TNF-α produced by the tumor-conditioned macrophage media. Incubation of both OSCC cell lines with PL significantly decreased the secretion of TNF-α and IL-1. Inherently, increased expression of IL-10, an immunosuppressive cytokine, was observed in macrophages conditioned with both SCC9 and LN1-tumoral media treated with PL. This cytokine has been reported to be upregulated in the saliva of patients with OSCC (Aziz et al., 2015); therefore, it is interesting that PL may downregulate genes or molecular pathways associated with the inhibition of IL-10. Taken together, these observations suggest that PL has a potential tumor immunoregulatory response, favoring an anti-inflammatory phenotype.

Notably, the microenvironment of oral cancer contains various transcription factors and inflammatory mediators that can induce proliferation and EMT, thereby promoting tumor invasion and metastasis. Neoplastic transformation causes changes in local cytokine production. Inflammatory cells present in tumor tissues may contribute to the development and maintenance of cancer *via* the release of mediators that regulate cell survival, proliferation, metabolism, angiogenesis, and tissue remodeling. Among these inflammatory mediators are the inflammatory cytokines IL-1 and IL-6, and tumor necrosis factor (TNF-α) (Bosch et al., 2015). Further, this process alters the expression of transforming growth factor-b (TGF-β), which, before mentioned, is the predominant regulator of the MMPs that remodel the ECM for tumor dissemination.

In addition, the immunomodulatory effect of PL on certain lymphocyte subsets and cytokines has already been described in the literature,(Sempere-Ortells et al., 2002; Sánchez-Rodríguez et al., 2018), showing the ability of PL to stimulate the proliferation and activation of T and natural killer lymphocytes, as well as an important downregulating effect on CD11, CD18, and CD62-L adhesion molecules, both on peripheral blood mononuclear cells and U-937 and HL-60 cell lines. Similarly, another study reported that PL has a modulatory effect on the *in vitro* production and release of cytokines by peripheral blood mononuclear cells (PBMNc) in healthy subjects (Sempere-Ortells et al., 2002). They also showed that at effective *in vivo* doses, PL could stimulate PBMNc proliferation, and delay IL-1beta secretion, and increase at the same time IL-2, IL-10, and IFN-γ levels.

Philips et al. also reported that PL inhibited TGF-β expression in melanoma cells (Philips et al., 2009a). PL demonstrated dual protective effects on the ECM *via* the inhibition of ECM proteolytic enzymes and stimulation of structural ECM collagens. These authors suggested that the effects of PL on melanoma cells could be partly attributed to the PL cell-specific regulation of TGF-β expression, and partly *via* its antioxidant properties (Philips et al., 2009a). Gonzalez et al. (2010) provided mechanistic insights into the PL extract as an oral and topical photoprotective agent. They also concluded that PL extract has the potential to counteract the upregulation of TGF-β during carcinogenesis *via* its antioxidant and anti-inflammatory properties.

To gain insights into the antitumor activity of PL *in vivo*, we generated a 4-NQO oral carcinogenesis model to represent human alcohol and tobacco abuse. The aim was to evaluate therapeutic efficacy of this potential polyphenolic natural plant in the orthotopic and immunocompetent 4-NQO mouse model. Animals chemically induced for 18 weeks were subjected to PL treatment for up to 20 weeks to determine the role of this herbal extract in preventing oral cancer and to investigate the putative molecular mechanisms by which PL could prevent oral tongue lesions.

The 4-NQO oral carcinogenesis orthotopic murine model, which faithfully reproduces the initiation and progression of human OSCC, offers an opportunity for a variety of preclinical studies. The model has been widely used in studies of oral carcinogenesis (Razzo et al., 2020; Bouaoud et al., 2021; Zigmundo et al., 2022) in cancer prevention research (Wang et al., 2017), mechanistic and chemopreventive studies of oral cancer (Tang et al., 2014; Shi et al., 2021), and for screening of therapeutic efficacy of novel drugs (de Oliveira et al., 2017; Siddappa et al., 2017; Ludwig et al., 2019). The advantage of using chemical induction in the process, with 4NQO as an inducing/promoting factor, is that it has a well-known mechanism of action: the generation of reactive oxygen (ROS) and nitrogen (ERN) species, such as superoxide radicals, hydrogen peroxide, and nitric oxide, which induce intracellular oxidative stress ROS and ERN, causing direct damage to macromolecules, including DNA, mainly by binding guanine residues, leading to the formation of adducts (Bouaoud et al., 2021). ROS participate in three stages of the carcinogenic process:1. During initiation, it causes genetic damage through a direct effect on the DNA or by activating other factors; 2. In the promotion stage, it favors the proliferation of malignant cells by inhibiting the mechanisms of immune control and promoting genomic instability; 3. Finally, it promotes the progression and dissemination of tumors by promoting protease release and angiogenesis (Bosch et al., 2015). This damage is similar to that caused by carcinogens present in tobacco, which is the main risk factor for oral cancer.

In our experiments, we observed lingual lesion formation in all mice analyzed, ranging from mild, moderate, and severe dysplastic lesions to well-differentiated carcinomas. It is worth mentioning that such variations may also reflect individual levels of carcinogens and PL ingestion by animals. We showed that animals treated with PL lesions were punctual, with a predominance of mild to moderate dysplasia, whereas in animals not treated with PL severe lesions, OSCCs were significantly higher. Tongue lesion tissue samples from mice not treated with PL showed remarkable Ki-67 staining. In fact, in the 4NQO-induced oral carcinogenesis experimental model, tumor progression was associated with the proliferative fraction of the tumor (cycling cells Ki-67 positive) and tumor differentiation (Alves et al., 2016) (Ref). Other studies have also shown that immunohistochemical expression of the ki-67 protein is correlated with the potential proliferation of malignant oral tumors (Warnakulasuriya, 2010; Alves et al., 2016). Notably, we observed a trend towards a decrease in the survival of mice not treated with PL (data not shown), which had more severe lesions, probably caused by the weakness of these animals already at an advanced age, the toxicity of the chemical carcinogen administered, and the progressive evolution of the disease due to the absence of the phytotherapeutic that appears to inhibit the progression of more severe lesions, compared to the animals treated with PL.

For the PL toxicological investigation of the animals (Murbach et al., 2015), we conducted a study on pure isolated preparations of a few of the eight identified phenolic compounds (considered to be the active antioxidant constituents) and concluded that no genotoxic activity of PL was identified in the *in vivo* micronucleus assay, together with the negative results observed in the bacterial reverse mutation and *in vitro* mammalian chromosomal aberration tests, assessing the safety of this botanical extract containing the eight different interacting phenolic compounds at low concentrations. This study was particularly important because it assessed full compliance with international protocols and provided a robust safety evaluation of PL owing to the inclusion of hematological and clinical chemistry evaluations, full gross and histopathological examinations, evaluation of relative and absolute organ weights, and inclusion of appropriate statistical analyses (Murbach et al., 2015). In addition, the low occurrence of adverse events observed in clinical trials involving PL and the history of consumption of PL as a commercial extract for more than 30 years, as well as the long history of traditional use without safety concerns, support the conclusions of the authors that provide meaningful evidence in consideration of the safety of consumption of PL by humans.

Finally, to explore the molecular outcome of PL modulation *in vivo* and corroborate our *in vitro* results, we sought to verify the gene expression of canonical proliferation- and EMT-associated pathways. Our results showed that the proliferative markers Bcl2 and Pcna were significantly downregulated in tongue dysplastic tissues and tongue carcinomas in animals treated with PL compared to non-treated animals. Recently, Pansini et al. (2021) reported that BCL2 plays a role in oral tumorigenesis and represents a promising biomarker that can recognize mesenchymal phenotype induction during the transition from non-malignant cells to tumor cells. PCNA has a dual function in that it is involved in DNA replication as well as DNA repair, both of which are important during cancer progression. We observed that in our 4NQO-induced oral carcinogenesis model, PL treatment was associated with a significantly lower ratio of cancer formation (23% vs. 55%) and a lower proportion of Ki67 positive cells, and Pcna expression in the lesion. The analysis of EMT markers in these lesions showed that animals treated with PL had significantly increased E-cadherin expression in OSCC and more severe lesions than mild and moderate dysplasia, compared to animals not treated with PL. In contrast, N-cad and Vim levels were significantly decreased in the lesions of animals treated with PL. These *in vivo* results corroborate our *in vitro* data, demonstrating that PL is an excellent herbal medicine for the control of oral cancer progression.

Although our study is the first to show the effects of PL in an *in vivo* oral carcinogenesis model, a previous study has shown that PL also has beneficial effects when administered orally. These animals were fed ∼300 mg/kg/day of PL extract or vehicle in their drinking water for 10 days, exposed to UV radiation, and evaluated for cyclooxygenase-2 (Cox-2) expression by western blot analysis. Upregulation of Cox-2 is associated with UV-induced skin cancer (Elmets et al., 2010; Müller-Decker, 2011). Cox-2 levels were 4-times lower in PL-fed mice at 48 h and 5-times lower at 72 h than in vehicle-fed mice. Other beneficial effects of PL include a significant decrease in UV-induced inflammation, including a 60% decrease in neutrophil infiltration into the skin at 24 h and a 50% decrease in macrophages in the skin at 24 and 48 h (Zattra et al., 2009).

Overall, PL is a safe and well-tolerated phytochemical across many studies, and the level of evidence for its clinical usefulness is strongest for several skin conditions (Searle et al., 2022). There are a growing number of patients in the cancer-associated field demonstrating a willingness to complement standard therapeutic regimens with natural health products, and general knowledge of the evidence-based data, risks, and benefits of these alternative treatments is helpful when counseling these patients. We showed that PL intake may be beneficial in preventing oral cancer progression; thus, phytochemical studies on the relevance of the antitumor effect of PL should be given more importance.

## Conclusion

These data demonstrated that PL has potential antitumor effects, particularly in EMT-associated oral carcinogenesis. As the early management of oral premalignant lesions are very important to their outcomes, our results may help to improve and develop preventive strategies for OSCC.

Although some chemoprevention strategies have been showed to potentially prevent OSCC, to date, no prevention strategy addressing earlier stages of oral cancerization is considered standard of care. Our data attempts to provide histological, molecular, and cellular characterization of premalignant lesions treated with a potent antitumor phytotherapy during their evolution into invasive cancers and is an important effort toward that goal. Preventing malignant transformation has also the potential to improve patient outcome. Thus, preclinical and clinical PL antitumor trials involving oral administration to patients with OSCC are warranted.

## Data Availability

The original contributions presented in the study are included in the article/Supplementary Material, further inquiries can be directed to the corresponding author.
