# Supplementary material for: Polypodium leucotomos targets multiple aspects of oral carcinogenesis and it is a potential antitumor phytotherapy against tongue cancer growth
Source: Front Pharmacol. 2023 Jan 5;13:1098374. doi: 10.3389/fphar.2022.1098374 (PMC9849903; doi:10.3389/fphar.2022.1098374)
Supplement: Supplementary file 2 [file Table2.docx]

**Supplementary Table 2**. Sequence of primers used for quantitative RT-PCR analysis of mouse and human genes.

| **Species** | **Gene** | **Forward (5’-3’)** | **Reverse (5’-3’)** |
| --- | --- | --- | --- |
| **Human** | **E-CAD** | ACAGCCCCGCCTTATGATT | TCGGAACCGCTTCCTTCA |
|  | **N-CAD** | TGGGAATCCGACGAATGG | CGTACGGCGCTGGGTATC |
|  | **VIM** | GGCTCGTCACCTTCGTGAAT | TCAATGTCAAGGGCCATCTTAA |
|  | **MMP-2** | TGATCTTGACCAGAATACCATCGA | GGCTTGCGAGGGAAGAAGTT |
|  | **MMP-1** | GGTGATGAAGCAGCCCAG | CAGTAGAATGGGAGAGTC |
|  | **TIMP-1** | TGCACCTGTGTCCCACCCCACCCACAGACG | GGCTATCTGGGACCGCAGGGACTGCCA GGT |
|  | **TIMP-2** | CCGAATTCTGCAGCTGCTCCCCGGTGCACCCG | GGAAGCTTTTATGGGTCCTCGATGTCGAG |
|  | **TGF-b** | *CAATTCCTGGCGATACCTCAG* | *GCACAACTCCGGTGACATCAA* |
|  | **TNF-a** | *GAGGCCAAGCCCTGGTATG* | *CGGGCCGATTGATCTCAGC* |
|  | **INOS** | *AGGGACAAGCCTACCCCTC* | *CTCATCTCCCGTCAGTTGG* |
|  | **B-ACT** | TCAGAAGGACTCCTATGTGG | TCTCTTTGATGTCACGCACG |
| **Mouse** | **Cdh1** | GGTTTTCTACAGCATCACCG | GCTTCCCCATTTGATGACAC |
|  | **Cdh2** | TGAAACGGCGGGGATAAAGAG | GGCTCCACAGTATCTGGTTG |
|  | **Vim** | CGGCTGCGAGAGAAATTGC | CCACTTTCCGTTCAAGGTCAAG |
|  | **Twist** | CTAGAGACTCTGGAGCTGGATAACTAAAAA | CGACCTCTTGAGAATGCATGCATGAAAAA |
|  | **Pcna** | TGC TCT GAG GTA CCT GAA CT | TGC TTC CTC ATC TTC AAT CT |
|  | **Bcl2** | GTGGATGACTGAGTACCT | CCAGGAGAAATCAAACAGAG |
|  | **B-actin** | CAT GTT TGA GAC CTT CAA CAC CCC | GCC ATC TCC TGC TCG AAG TCT AG |
