# Supplementary material for: Polypodium leucotomos targets multiple aspects of oral carcinogenesis and it is a potential antitumor phytotherapy against tongue cancer growth
Source: Front Pharmacol. 2023 Jan 5;13:1098374. doi: 10.3389/fphar.2022.1098374 (PMC9849903; doi:10.3389/fphar.2022.1098374)
Supplement: Supplementary file 3 [file Table3.DOCX]

**Supplementary Table 3.** Distribution of Ki-67 expression pattern among lesions of tongue of animals treated and non-treated with PL analyzed by chi-square test

| **Outcome** | **+4NQO+PL (27)** | **+4NQO-PL (33)** | **P value** |
| --- | --- | --- | --- |
| **Ki-67 Low** | 23 (85,2%) | 13 (39,4%) | P<0.0001 |
| **Ki-67 High** | 4 (14,8%) | 20 (60,6%) |  |
